# Changes to physical activity behavior during the COVID-19 pandemic and their associated factors: a cross sectional survey of Mexican women

**DOI:** 10.1186/s12905-023-02393-1

**Published:** 2023-05-11

**Authors:** Catherine Stratton, Maryam Fourtassi, Ioana Ramia, Uma Pandiyan, Rory Cooper, Abderrazak Hajjioui, Andrei Krassioukov, Mark D. Peterson, Joseph K Balikuddembe, Angela Palomba, Bo-Young Hong, Deo Rishi Tripathi, Yetsa A Tuakli-Wosornu, Laura Paulina Muñoz Velasco

**Affiliations:** 1 Department of Epidemiology, Dalla Lana School of Public Health, University of Toronto, Toronto, Canada; 2 Laboratory of Life and Health Sciences, Faculty of Medicine and Pharmacy of Tangier, Abdelmalek Essaâdi University, Tetouan, Morocco; 3 University of New South Wales, Sydney, Australia; 4 Qatar Rehabilitation Institute, Hamad Medical Corporation, Doha, Qatar; 5 Department of Veteran Affairs, Human Engineering Research Laboratories (HERL), Pittsburgh, US; 6 School of Health and Rehabilitation Sciences, University of Pittsburgh, Pennsylvania, USA; 7 Clinical Neuroscience Laboratory, Faculty of Medicine, Pharmacy and Dentistry, University Sidi Mohamed Ben Abdellah Fez, Fez, Morocco; 8 Department of Physical Medicine and Rehabilitation, Teaching University Hospital Hassan II of Fez, Atlas, Morocco; 9 International Collaboration on Repair Discoveries, Faculty of Medicine, University of British Columbia, Vancouver, Canada; 10 Division of Physical Medicine and Rehabilitation, Department of Medicine, University of British Columbia, Vancouver, Canada; 11 G.F. Strong Rehabilitation Centre, Vancouver, Canada; 12 Department of Physical Medicine and Rehabilitation, Michigan Medicine, University of Michigan, Ann Arbor, USA; 13 Institute for Disaster Management and Reconstruction, Sichuan University and Hong Kong Polytechnic University, Chengdu, China; 14 Center on Disability Studies (CDS), University of Hawaii, Monoa, USA; 15 Department of Mental and Physical Health and Preventative Medicine, University of Campania “Luigi Vanvitelli”, Naples, Italy; 16 Department of Rehabilitation Medicine, St. Vincent’s Hospital, The Catholic University of Korea, Seoul, Korea; 17 Department of Physical and Rehabilitation, Hamdard Institute of Medical Science and Research, New Delhi, India; 18 Department of Amputee Rehabilitation, National Institute of Rehabilitation Luis Guillermo Ibarra Ibarra, Mexico City, Mexico; 19 Department of Social and Behavioral Sciences, Yale School of Public Health, Yale University, New Haven, USA

**Keywords:** COVID-19, Physical activity, Women, Mexico, Perceived functioning level

## Abstract

**Background:**

On March 24, 2020, the Mexican Government established social distancing measures to address the outbreak of the COVID-19 pandemic. The resulting home confinement affected daily lifestyle habits such as eating, sleeping, and physical activity (PA). The objectives of this study were to determine changes in PA behaviors among Mexican women due to the COVID-19 pandemic and to assess potential factors associated with these changes.

**Methods:**

This was a cross-sectional study based on an anonymous online questionnaire developed by the Task Force on Physical Activity for Persons with Disabilities (PAPD) within the International Society of Physical and Rehabilitation Medicine (ISPRM). Descriptive, quantitative statistics were used for data analysis. A Chi-squared (χ²) test was used to explore associations between dependent and independent variables.

**Results:**

A total of 1882 surveys were completed. Among the respondents, 53.3% declared that their PA was reduced during the pandemic, 26.6% reported similar PA behavior, and 20.1% declared that their PA had increased during the pandemic. Lower PA behavior during the pandemic was associated with lower education levels, stricter pandemic constraints, obesity, and lower self-perceived functioning levels. A statistically significant association between poorer self-perceived mental health and decreased PA behaviors was also found. Respondents who were younger, self-perceived as unimpaired, not overweight, and whose income was not impacted by COVID-19 were associated with higher levels of reported physical and mental health.

**Conclusions:**

The study results identify disparities experienced in PA behavior during the COVID-19 pandemic among Mexican women and highlights the need for social support for PA participation.

## Background and significance of the study

On March 24, 2020, the Mexican Government established social distancing measures to control the outbreak of the COVID-19 pandemic [1]. Schools from all academic levels were closed, public events were canceled, and non-essential economic activities were suspended, causing significant changes to daily life for millions of families [1].

COVID-19 restrictions exacerbated gender differences with women being more affected in economic and social spheres. In addition to representing 70% of health and social-service workers worldwide, women also frequently take full responsibility for household members [2]. COVID-related school closings in Mexico caused nearly 25.5 million students to stay at home. More than 9 million households have at least one child under six years old, which significantly increased unpaid care workload usually performed by women [3]. Further, the confinement of COVID-19 restrictions, while necessary for virus containment, increased risks such as, violence against and burnout among women, which contributed to a physically, mentally and emotionally challenging socio-cultural environment [4].

Home confinement affected lifestyle habits such as eating, sleeping and physical activity (PA) [5]; physical and mental health became affected due to restricted access to health services, gyms and fitness centers, city parks and playgrounds, and other recreational spaces where group exercise usually takes place, causing a negative effect on PA engagement [6]. The ability to engage in recreation and PA can mediate stress and promote self-care, and therefore, can serve as an acute coping mechanism that provides additional positive benefits on physical and mental health [6]. On the other hand, reduced PA is associated with an increased risk of cardiovascular diseases, obesity, certain cancers, hypertension, type 2 diabetes, all-cause mortality, and can also lead to disability or long-term illness associated with these conditions. [7–13] In addition to these physical conditions, sedentariness may be associated with mental health challenges, including clinical depression and general anxiety disorder (GAD) [10–13].

Prior to the pandemic, estimates suggested that 29% of Mexican adults did not achieve 150 min/week of PA - the recommended health standard by the World Health Organization (WHO) and the American College of Sports Medicine [14]. Moreover, the Mexican population had a reported overweight and obesity prevalence of 72.5%, reflective of the effects of a sedentary lifestyle and expected to worsen with the pandemic restrictions [15].

Other, smaller studies, found a much larger proportion of participants had low levels of PA, with a significant gender gap (49% of women and 39% of men) [16]. Generally, women report a greater number of barriers to exercise than men and these barriers are associated with lower PA participation rates in pre-pandemic studies. For example, lack of enjoyment, self-consciousness, and time constraints are frequently cited by women as barriers to being regularly physically active [6].

A systematic review and meta-analysis assessing the effects of the pandemic on PA behavior revealed heterogenous results but with a trend towards a significant decrease ​[17]. Six investigations reported results regarding differences between gender. Of these, one study found a significant reduction of PA in men only, one study found this reduction in women only, and one study detected a marginal difference, where women had a higher increase in moderate physical activity and walking than men [17]. The remaining three studies did not detect any differences or found that the pandemic had the same effect for both genders ​[17]​.

Despite the known benefits of PA on physical and mental health, the generally negative impact of the pandemic on participation in PA, and the recognized gender differences in participation, little is known about the effect of COVID-19 pandemic-imposed restrictions on Mexican women’s PA behavior, associated factors, and consequences for mental and physical health self-perception. This is important to understand given the context of a population already ranked relatively low in terms of participation in PA. The focus on women’s experiences is particularly important as studies suggest women’s societal roles and barriers on the other may contribute to potentially lasting negative outcomes for women. While this study focuses on Mexican women, results are likely to be applicable internationally, to other countries affected by the pandemic and associated restrictions. As such, we sought to address the question: If any exist, what were the changes to physical activity behaviors during the COVID-19 pandemic and their associated factors among a sample of Mexican women?

### Objectives

*This study will aim to address the following objectives*:

1. Describe changes in physical activity behaviors among participants due to COVID-19 restrictions;
2. Explore the potential factors associated with these changes; and,
3. Evaluate the potential association(s) between these changes on self-perceived physical and mental health.

## Methods

### Study design

We conducted a cross-sectional study as part of an international online survey designed to investigate global changes in lifestyle during the COVID-19 pandemic, by the Task Force on Physical Activity for Persons with Disabilities (PAPD) within the International Society for Physical and Rehabilitation Medicine (ISPRM).

### Population

All completed questionnaires from adult respondents (age > 18 years old) were included in the global database, and result haven been published in previous papers [18, 19]. We decided to do conduct the present analysis on Mexican women specifically from within the larger dataset due to the higher rate of responses among this demographic on the survey.

### Data collection

The survey was circulated between August and December in 2020, based on a snowball sampling strategy. Members of the Task Force, who were mostly physical medicine and rehabilitation specialists were asked to share the online questionnaire with their respective networks, patients, students, and social media communities and asked the respondents to also distribute the questionnaire to their respective circles of contacts.

The first section of the survey collected general sociodemographic data such as, sex, age, educational level, underlying health conditions, and employment status. Activity limitations and financial consequences from the COVID-19 pandemic restrictions were also assessed.

The second section assessed functioning and participation levels with the Washington Group Questions (WGQ) and the International Classification of Functioning, Disability and Health (ICF) which included six items relating to activities and participation: “Understanding and communication”; “Getting around/mobility”; “Self-care”; “Getting along with people”; “Life Activities”; and “Participation in Society” [20, 21]. Participants were asked to score questions on a scale of 0–4, with “0” representing no functioning and “4” representing complete functioning. Participants who self-scored every question as “4” were considered “not to have a disability” for the purposes of this study. By contrast, anyone who scored one or more questions as “0” were considered to have a “severe disability”. All other scoring combinations were classified as having “mild” to “moderate disability”.

Physical activity behaviors before and during the pandemic were assessed by asking participants how many days per week they engage in 30 min or more of PA. Physical activity was classified into three categories of performance: (1) low (< 2 days/week); (2) moderate (2–4 days/week); and (3) high (≥ 5 days/week).

Perceived physical and mental health were explored using two simple questions with 3 possible answers each; 1-“Generally, during the pandemic, I think my physical health and physical fitness has been: same as before, better than before, worse than before”. 2- “Generally, during the pandemic, I think my mental health and emotional wellness (i.e. anxiety, depression, sadness, connectedness) has been: same as before, better than before, worse than before”.

### Statistical analysis

As this is cross-sectional survey, a methodology that aims to capture as many people as possible, and with a population size that is unknown, a sample size estimation was not preformed *a priori.*

Descriptive quantitative statistics were used for data analysis. Changes in PA level and perceived physical and mental health during the pandemic were classified as “less than”, “unchanged” or “more than” before the pandemic. A Chi-squared (χ²) test was used to explore associations between dependent variables (levels of PA change during the pandemic) and independent variables (sociodemographic variables, health-related variables, disability and functioning levels, social aspects related to the pandemic). χ² was also used to evaluation associations between changes to PA behavior during the pandemic and perceived physical and mental health. All statistical tests were performed using SPSS 20.0. An *a piori* p-level of < 0.05 was set as the level of significance.

### Ethics

This research was submitted for review to Yale University’s Institutional Review Board’s (IRB) Human Research Protection Program and was deemed exempt by the IRB (45CFR46.104.). Participants were provided with a link to the survey and on the opening page of the survey, the purpose of the survey was described so that participants could make an informed decision whether to participate. Informed consent was obtained from participants through the first question of the survey which asked for participants’ consent. Data confidentiality was respected through anonymizing results. All methods performed in this study were performed in accordance with the Declaration of Helsinki.

## Results

### Demographics and disability level

Within the study period, 1882 women from Mexico completed the entire survey. Demographic data are shown in Table 1.

**Table 1:** Demographics and health issues

	All participants N (%)
**Age groups**	
Under 40	1182 (62.8)
Over 40	700 (37.2)
**Education**	
Postgraduate education	1372 (72.9)
Undergraduate education	487 (25.9)
High school education or less	23 (1.2)
**Professional Activity**	
Employed or student	1692 (89.9)
Unemployed or retired	190 (10.1)
**Chronic Disease**	
Yes	573 (30.4)
No	1309 (69.6)
**Overweight/Obesity**	
Yes	468 (24.9)
No	1414 (75.1)
**Smoking**	
Yes	216 (11.5)
No	1666 (88.5)

Among the respondents, 835 women perceived themselves as having some degree of disability in functioning and engagement (44.4%); of whom, 401 were classified as having mild-moderate disability (21.3%) and 434 as having severe disability (23.1%). Details about the degrees of self-perceived functioning levels, graded based on activity and participation items (Table 2).

**Table 2:** Perceived functioning level categories and assessment degrees

	Full functioning	Moderate functioning	Some functioninsg	No functioning
**Activity and Participation**	N (%)	N (%)	N (%)	N (%)
Understanding and communication	1674 (88.9)	182 (9.7)	23 (1.2)	3 (0.2)
Getting around/ Mobility	1640 (87.1)	186 (9.9)	49 (2.6)	7 (0.4)
Self-care	1824 (96.9)	43 (2.3)	12 (0.6)	3 (0.2)
Life Activities	1704 (90.5)	137 (7.3)	39 (2.1)	2 (0.1)
Participation in Society	1380 (73.3)	183 (9.7)	200 (10.6)	119 (6.3)
Getting along with people	1337 (71.0)	224 (11.9)	253 (13.4)	68 (3.6)

### Physical activity before the pandemic

Before the pandemic, only 19.5% of participants engaged in at least 30 min of PA for more than four days per week (“high PA profile”), whereas 38.5% of participants were physically active 2–4 days per week (“moderate PA profile”), and 42.0% of participants were physically active fewer than two days per week (“low PA profile”). Non-working women were more active than those who were employed or students.

As shown in Table 3, no statistically significant association was found between the pre-pandemic PA profiles and age, education, self-perceived functioning level, or the presence of a chronic disease. The analysis did reveal, however, that obese/overweight and occupation status were significantly associated with low PA profiles before the pandemic (p < 0.001 and p = 0.048, respectively).

**Table 3:** Physical Activity profiles before pandemic and associated factors

	Physical Activity Behavior			
	Low	Moderate	High	P-value
	N (%)	N (%)	N (%)	
**Age groups**				0.326
Under 40 (1182)	507 (42.9)	455 (38.5)	220 (18.6)	
Over 40 (700)	281 (40.1)	271 (38.7)	148 (21.1)	
**Education**				0.462
High school education or less/Undergraduate education (510)	225 (44.1)	187 (36.7)	98 (19.2)	
Postgraduate education (1372)	563 (41.0)	539 (39.3)	270 (19.7)	
**Professional Activity**				**0.048**
Employed or student (1692)	720 (42.6)	653 (38.6)	319 (18.9)	
Unemployed or retired (190)	68 (35.8)	73 (38.4)	49 (25.8)	
**Chronic Disease**				0.693
Yes (573)	247 (43.1)	213 (37.2)	113 (19.7)	
No (1309)	541 (41.3)	513 (39.2)	255 (19.5)	
**Overweight/Obesity**				**< 0.001**
Yes (468)	228 (48.7)	176 (37.6)	64 (13.7)	
No (1414)	560 (39.6)	550 (38.9)	304 (21.5)	
**Functioning and engagement perception**				0.729
No disability (1047)	429 (41.0)	403 (38.5)	215 (20.5)	
Mild-Moderate (401)	177 (44.1)	152 (37.9)	72 (18.0)	
Severe (434)	182 (41.9)	171 (39.4)	81 (18.7)	

### Perceived physical health during the pandemic

During the pandemic, 897 (47.7%) of the participants perceived their physical health as worse than before the pandemic, 616 (32.7%) perceived their physical health as unchanged, and 369 (19.6%) of participants declared an improvement (Table 4).

**Table 4:** Physical Activity behavior change during pandemic and associated factors

	Less	Unchanged	More	P-value
	N (%)	N (%)	N (%)	
**Age groups**				0.254
Under 40 (1182)	647 (54.7)	307 (26.0)	228 (19.3)	
Over 40 (700)	356 (50.9)	194 (27.7)	150 (21.4)	
**Education**				**0.013**
High school education or less/Undergraduate education (510)	270 (52.9)	156 (30.6)	84 (16.5)	
Postgraduate education (1372)	733 (53.4)	345 (25.1)	294 (21.4)	
**Professional Activity**				0.179
Employed or student (1692)	899 (53.1)	444 (26.2)	349 (20.6)	
Unemployed or retired (190)	104 (54.7)	57 (30.0)	29 (15.3)	
**Chronic Disease**				0.606
Yes (573)	305 (53.2)	146 (25.5)	122 (21.3)	
No (1309)	698 (53.3)	355 (27.1)	256 (19.6)	
**Overweight/Obesity**				**0.036**
Yes (468)	254 (54.3)	138 (29.5)	76 (16.2)	
No (1414)	749 (53.0)	363 (25.7)	302 (21.4)	
**Functioning and engagement perception**				**0.001**
No disability (1047)	524 (50.0)	283 (27.0)	240 (22.9)	
Mild-Moderate (401)	222 (55.4)	101 (25.2)	78 (19.5)	
Severe (434)	257 (59.2)	117 (27.0)	60 (13.8)	
**Physical activity behavior before pandemic**				**< 0.001**
Low (788)	222 (28.2)	343 (43.5)	223 (28.3)	
Moderate (726)	534 (73.6)	79 (10.9)	113 (15.6)	
High (368)	247 (67.1)	79 (21.5)	42 (11.4)	
**Pandemic restrictions**				**0.009**
Quarantine (896)	476 (53.1)	233 (26.0)	187 (20.9)	
Lockdown (433)	243 (56.1)	94 (21.7)	96 (22.2)	
Social distancing (553)	284 (51.4)	174 (31.5)	95 (17.2)	
**Pandemic impact on income**				0.350
Reduced income/Loss of Job (763)	422 (55.3)	195 (25.6)	146 (19.1)	
No Impact on income (1119)	581 (51.9)	306 (27.3)	232 (20.7)	
**Perceived physical health**				**< 0.001**
Worse (897)	671 (74.8)	188 (21.0)	38 (4.2)	
Same (616)	268 (43.5)	249 (40.4)	99 (16.1)	
Better (369)	64 (17.3)	64 (17.3)	241 (65.3)	
**Perceived mental health**				**< 0.001**
Worse (1389)	801 (57.7)	356 (25.6)	232 (16.7)	
Same (361)	171 (47.4)	107 (29.6)	83 (23.0)	

### Perceived mental health during the pandemic

Most respondents (1389, 73.8%) reported a worsening in their self-perceived mental health during the pandemic. Another 361 (19.2%) respondents reported that their mental health remained unchanged from before and 132 (7.0%) declared an improvement in their mental health.

### Physical activity behavior change during pandemic and associated factors

When asked to estimate the change(s) in PA participation during the pandemic compared to before, 53.3% of participants declared that their PA was reduced, 26.6% reported unchanged PA, and 20.1% declared that their PA had increased during the pandemic.

Significantly reduced PA behaviors were associated with higher education level, more extensive pandemic-imposed restrictions (lockdown and quarantine), being obese/overweight, and worse self-perceived functioning (all p < 0.05). Also, poorer self-perceived physical and mental health and lower PA participation prior to the pandemic were statistically significantly associated with a decrease of PA during pandemic (p < 0.001). Factors found to be associated with the PA behavior change during the pandemic are described in Table 4.

## Discussion

To our knowledge, this is the first paper assessing the COVID-19 pandemic’s related changes to PA behaviors and possible associated factors, among Mexican women.

Our study found that prior to the pandemic only 19.5% of Mexican women met the PA recommendations established by the WHO before the pandemic. It is possible that a lack of available time and fatigue acted as barriers to PA behaviors among working women [6]. Obese and overweight women were less active, reiterating the well-established link between physical inactivity and overweight status [13].

We found that PA behaviors were negatively affected during the pandemic overall. More than a half of our participants declared that their PA level was reduced. These data are consistent with those from other studies, including the Mexican National Health and Nutrition Survey of 2020 [22], a global study including 3079 participants from 58 countries [23], and a Brazilian study carried out among healthcare professionals during the pandemic [24]. Even though the WHO provided guidance on how to stay active and reduce sedentary behavior while at home to individuals in self-quarantine, PA has been significantly reduced worldwide during the COVID-19 pandemic [25].

Reduced PA behaviors during the pandemic were associated with more extensive pandemic restrictions (e.g., lockdown, quarantine), being overweight/obese, lower self-perceived functioning, and among less-education women. Our results are consistent with the GLOBE study findings where higher education was associated with greater PA engagement [26].

Care required by non-hospitalized persons infected with COVID-19 and young children due to pandemic restrictions, such as closures to child care centers and schools, was commonly provided by women, in addition to their usual professional and domestic work. This circumstance increased their workloads, and for some, their physical and emotional stresses, thereby potentially adding barriers for PA engagement [4, 6, 27, 28].

People living with disabilities, who represent 16.5% of Mexico’s population [29], frequently encountered numerous barriers to participate in sports, exercise, and PA behaviors before the pandemic. Independent of any physical or cognitive impairments that might be experienced secondary to disability(ies), social barriers were widely experienced, including limited accessibility to exercise facilities, lack of suitable transportation to such facilities where they did exist, lack of social awareness about accessibility, and reduced financial resources [30]. The pre-existing gap in access to PA for people living with disabilities has widened during the COVID-19 pandemic [30]. While a great number of digital fitness resources were devised during the COVID-19 crisis, the majority has been geared towards people without impairments or disabilities [31]. This phenomenon was observed in the study’s findings where the relationship between disability status and change in PA behaviors was statisitically significant and a greater proportion of women with severe disability showed a decrease in PA during the pandemic than the other disability status groups.

In our study, a strong association was found between reduced PA behavior during pandemic and poorer self-perceived physical and mental health self-perception, and vice versa. This is similar to international studies that found better mental health scores among women who were able to remain or become more active during the pandemic [6, 32]. A Canadian cross-sectional study displayed that women who were able to remain active or become more active during the pandemic had better mental health scores [6]. Similar results were found in an Italian study where a significant positive correlation was found between the variation of PA and mental well-being, suggesting that reduction of total PA is related to worse status of psychological well-being [32]. Strikingly, this correlation appeared to be stronger in women [32].

Our study’s finding that poor self-perceived functioning was a predictor of poorer physical and mental health perception as well, was consistent with the global study data [23]. Even before the pandemic, people living with disabilities faced many challenges to engage fully in public life such as, limited access to health services, fewer job opportunities, and greater resulting unemployment. With the pandemic, this population became even more vulnerable, as their underlying conditions put them at a greater risk of infection and noncommunicable disease. Moreover, their health care needs were not being fully met due to the significant strain the pandemic had on the healthcare system, and COVID-imposed restrictions made participation in PA an even greater challenge [33].

The high percentages of the women who reported self-perceived worsened health during the pandemic, (47.7% on physical health and 73.8% on mental health), are expected findings due to possible isolation from normal social life and boredom, death of family or friends from the COVID-19 virus, fear of infection, frustration with inadequate information, and financial loss. Other studies have suggested that the COVID-19 pandemic and associated restrictions had led to lower levels of physical and mental health in the overall population compared to pre-pandemic levels [34, 35], and our findings further confirm this pattern is also observed in a sample of Mexican women.

The present study yields many implications for future policy-making decisions. It is of concern that the pandemic caused substantial declines in PA in many population categories with the possibility that many adults might never again achieve their pre-pandemic PA levels. This would imply an expected increase in morbidity and mortality in the upcoming years, as these parameters are directly associated with low PA levels ​[36].

### Limitations

The main limitation of this study was that the questionnaires are self-reported, which can introduce bias. However, in the context of conducting research under social isolation, this was a feasible research method. Further, this allowed a greater sample of people to be reached than if scientists administered the survey themselves in their respective clinics or institutions.

Snowball sampling over social media was used among Mexican female doctors which introduced selection bias, explaining the overrepresentation of young and highly educated women in our study. People needed to have access to the internet and connected technology (e.g., smartphone, tablet, computer) to complete the survey.

A higher proportion of respondents perceived themselves as having some kind of impairment in functioning and engagement than the officially reported proportion of people living with disabilities by the National Institute of Statistics and Geography in Mexico (44.4% vs. 16.5%) [4]. This may be because the questions inquired about function, although based on the ICF activity, were interpreted as impaired function in the social sphere. This might be explained by the pandemic’s social restrictions which could have had a greater effect on the circumstances of women respondents who previously worked in in-person settings with other people.

We do not have specific information about the region/state in Mexico where the participants lived because the survey was originally intended to be distributed globally. As we received a significant proportion of Mexican participants, we decided to focus and analyze data from this significant group of women.

## Conclusions

Mexican women, particularly those who identify as having a disability, reported poorer PA behaviors and self-perceived mental and physical health during the COVID-19 pandemic. These are worrisome findings that indicate the need to advocate for support and provide community opportunities for PA to reduce the negative impacts that lacking these resources have had on physical and mental health. Special attention should be paid to professional women and those living with disabilities.

It is important that further studies explore the impacts of the emotional and physical burdens faced by professional women who had increased caregiving responsibilities in the home setting in addition to their paid work. More equitable conditions that increase women’s opportunities for self-care, like PA, that will enhance social support and coping strategies to reduce stress and improve physical health are paramount. We believe that it is also crucial to change the public dependency on gym-based workouts and explore alternative PA options for the home-setting which can help women restore their mental and physical health who have been more likely to stay in the home-setting during isolation due to the pandemic. Lessons from the COVID-19 pandemic’s social restrictions can be applied to future research to lessen the burden on women during such crises so that their physical and mental well-being can be maintained or even improved, rather than worsened. Similarly, the pandemic highlighted and exposed the structural barriers to PA resources people living with disabilities face which need to be addressed.

## Data Availability

The datasets generated and/or analysed during the current study are not publicly available because the database is still in use by the authors, but are available from the corresponding author on reasonable request.

## References

[1] María O, Carmen DEL, Cordero S, Gobernación S De. Secretaría de Gobernación. 2020; Available from https://www.dof.gob.mx/nota_detalle.php?codigo=5590339&fecha=24/03/2020#gsc.tab=0

[2] Boniol M, Mcisaac M, Xu L, Wuliji T, Diallo K, Campbell J. Gender equity in the health workforce: Analysis of 104 countries. 2019 [; Available from: http://apps.who.int/bookorders.

[3] INMUJERES. COVID-19 y su impacto en números desde la perspectiva de género. 2019;10. Available from: https://www.gob.mx/cms/uploads/attachment/file/543160/Covid19-cifrasPEG.pdf

[4] El impacto del COVID-19 en la salud de las mujeres. Available from: https://ibero.mx/prensa/cae-por-covid-19-50

[5] Di Renzo L, Gualtieri P, Pivari F, Soldati L, Attinà A, Cinelli G (2020). Eating habits and lifestyle changes during COVID-19 lockdown: an italian survey. J Transl Med.

[6] Nienhuis CP, Lesser IA (2020). The impact of COVID-19 on women’s physical activity behavior and mental well-being. Int J Environ Res Public Health.

[7] Li T, Wei S, Shi Y, Pang S, Qin Q, Yin J, et al. The dose-response effect of physical activity on cancer mortality: findings from 71 prospective cohort studies. Available from: 10.1136/bjsports-2015-094927 10.1136/bjsports-2015-094927 26385207

[8] Kujala UM, Kaprio J, Sarna S, Koskenvuo M. Relationship of Leisure-Time Physical Activity and Mortality The Finnish Twin Cohort. Available from: https://jamanetwork.com/10.1001/jama.279.6.440 9466636

[9] Fishman EI, Steeves JA, Zipunnikov V, Koster A, Berrigan D, Harris TA (2016). Association between objectively measured physical activity and mortality in Nhanes. Med Sci Sports Exerc.

[10] García-Tascón M, Sahelices-Pinto C, Mendaña-Cuervo C, Magaz-González AM (2020). The impact of the covid-19 confinement on the habits of pa practice according to gender (Male/female): spanish case. Int J Environ Res Public Health.

[11] Mikkelsen K, Stojanovska L, Polenakovic M, Bosevski M, Apostolopoulos V (2017). Exercise and mental health. Maturitas.

[12] Forouzanfar MH, Afshin A, Alexander LT, Biryukov S, Brauer M, Cercy K (2016). Global, regional, and national comparative risk assessment of 79 behavioural, environmental and occupational, and metabolic risks or clusters of risks, 1990–2015: a systematic analysis for the global burden of Disease Study 2015. Lancet.

[13] Pedersen BK, Saltin B (2015). Exercise as medicine - evidence for prescribing exercise as therapy in 26 different chronic diseases. Scand J Med Sci Sport.

[14] Secretaría de salud de México. Encuesta Nacional de Salud y Nutrición. Ensanut. 2018;1:47. Available from: https://ensanut.insp.mx/encuestas/ensanut2018/doctos/informes/ensanut_2018_presentacion_resultados.pdf

[15] Flores-Olivares LA, Cervantes-Hernández N, Quintana-Mendias E, Enriquez-Del Castillo LA (2021). Actividad física y estilo de vida sedentario en adultos, cambios durante el confinamiento por la pandemia de Covid-19. Salud Publica Mex.

[16] RICO-GALLEGOS CG, VARGAS G, POBLETE-VALDERRAMA FA, CARRILLO-SANCHEZ J, RICO-GALLEGOS J, MENA-QUINTANA B (2020). Hábitos de actividad física y estado de salud durante la pandemia por COVID-19. Rev Espac.

[17] Wunsch K, Kienberger K, Niessner C. Changes in physical activity patterns due to the COVID-19 pandemic : a systematic review and Meta-analysis. Vol. 19, International Journal of Environmental Research and Public Health. MDPI; 2022. 10.3390/ijerph19042250 PMC8871718 35206434

[18] Tuakli-Wosornu YA, Wang K, Fourtassi M, Stratton C, Muñoz-Velasco LP, Hajjioui A, Hong BY (2023). Impact of the COVID-19 pandemic on the Perceived Physical and Mental Health and healthy lifestyle behaviors of people with disabilities: a quantitative analysis of the International Community Survey. American Journal of Physical Medicine & Rehabilitation.

[19] Azzouzi S, Stratton C, Muñoz-Velasco LP, Wang K, Fourtassi M, Hong BY, Hajjioui A (2022). The impact of the COVID-19 pandemic on healthy lifestyle behaviors and perceived mental and physical health of people living with non-communicable diseases: an international cross-sectional survey. International Journal of Environmental Research and Public Health.

[20] Washington Group. Short Set of Questions on Disability. CDC National Center for Health Statistics. 2015. Available online: https://www.cdc.gov/nchs/washington_group/wg_questions.htm

[21] World Health Organization. International Classification of Functioning, Disability and Health: ICF; World Health Organization: Geneva, Switzerland, 2001. Available online: https://apps.who.int/iris/handle/10665/42407

[22] Shamah LT, Romero MM, Barrientos GT, Cuevas NL, Bautista AS, Colchero MA, et al. Encuesta Nacional de Salud y Nutrición 2020 sobre Covid-19. Resultados nacionales. Vol. 148. 2021. 148–162 p. Available from: https://ensanut.insp.mx/encuestas/ensanutcontinua2020/doctos/informes/ensanutCovid19ResultadosNacionales.pdf

[23] Hong BY, Stratton C, Muñoz-Velasco LP, Peterson M. Impact of the COVID-19 Pandemic on the Perceived Physical and Mental Health and Healthy Lifestyle Behaviors of People with Disabilities : Quantitative Analysis of the International Community Survey. In-press. 10.1097/PHM.0000000000002056 35687754

[24] Mota IA, de Oliveira Sobrinho GD, Morais IPS, Dantas TF (2021). Impact of COVID-19 on eating habits, physical activity and sleep in brazilian healthcare professionals. Arq Neuropsiquiatr.

[25] Mattioli AV, Coppi F, Gallina S. Importance of physical activity during and after the SARS-CoV‐2/COVID‐19 pandemic: a strategy for women to cope with stress. Eur J Neurol. 2021;(March):1–2. 10.1111/ene.14945 PMC8239911 34048097

[26] Droomers M, Schrijvers CTM, Mackenbach JP. Educational level and decreases in leisure time physical activity: predictors from the longitudinal GLOBE study. J Epidemiol Community Heal. 2001 Aug 1;55(8):562–8. 10.1136/jech.55.8.562 PMC1731951 11449013

[27] Guezmes A, Espinosa D, Bonaffe J, Vasconez A, Barba L. CARE IN LATIN AMERICA AND THE CARIBBEAN DURING THE COVID-19. TOWARDS COMPREHENSIVE SYSTEMS TO STRENGTHEN RESPONSE AND RECOVERY. Vol. v 1.1. 19.08.2020, Brief. 2020.

[28] Observatorio de Género y COVID-19. Cuidados - Género y Covid. Observatorio de Género y COVID-19. 2021. Available from: https://genero-covid19.gire.org.mx/tema/trabajo-de-cuidados/

[29] INEGI. Estructura De La Población 2000, 2010 Y 2020. Censo Poblac y Vivienda. 2021;1–3. Available from: http://censo2020.mx/

[30] Diaz R, Miller EK, Kraus E, Fredericson M. Impact of Adaptive Sports Participation on Quality of Life. 2019;(October). 10.1097/JSA.0000000000000242 31046012

[31] Stratton C, Kadakia S, Balikuddembe JK, Peterson M, Hajjioui A, Cooper R (2020). Access denied: the shortage of digitized fitness resources for people with disabilities. Disabil Rehabil.

[32] Maugeri G, Castrogiovanni P, Battaglia G, Pippi R, D’Agata V, Palma A, et al. The impact of physical activity on psychological health during Covid-19 pandemic in Italy. Heliyon. 2020 Jun 1;6(6). 10.1016/j.heliyon.2020.e04315 PMC7311901 32613133

[33] ÖZYILMAZ KÜÇÜKYAĞCI P (2020). Covid-19 and its Effect on People with Disabilities on Urban Life. Atlas J.

[34] Togni G, Puccinelli PJ, Costa T, et al. Factors Associated with Reduction in Physical Activity during the COVID-19 Pandemic in São Paulo, Brazil: An Internet-Based Survey Conducted in June 2020. *Int J Environ Res Public Health*. 2021;18(21):11397. Published 2021 Oct 29. 10.3390/ijerph182111397. 10.3390/ijerph182111397 PMC8583209 34769910

[35] Tuakli-Wosornu YA, Pandiyan U, Stratton C, Hwang Y, Hajjioui A, Muñoz-Velasco LP, Fourtassi M, Cooper R, Balikuddembe JK, Peterson M, Krassioukov A, Palomba A, Tripathi DR, Hong BY (2022). Perceived physical and Mental Health and Healthy Eating Habits during the COVID-19 pandemic in Korea. J Korean Med Sci.

[36] Joseph RP, Pituch KA, Guest MA, Maxfield M, Peckham A, Coon DW, et al. Physical activity among predominantly White Middle-Aged and older US adults during the SARS-CoV-2 pandemic: results from a National Longitudinal Survey. Front Public Health. 2021 Apr 13;9. 10.3389/fpubh.2021.652197 PMC8076643 33928065

